# Successful Treatment of Long-Standing Dermatillomania With Sertraline in a Rural Mexican Patient: A Case Report and Literature Review

**DOI:** 10.7759/cureus.94123

**Published:** 2025-10-08

**Authors:** Ricardo Cid-Puente, Paola V Rosales-Verduzco, Leticia Lizama-Rubio, Ana I Diaz de León-Guzmán

**Affiliations:** 1 Department of Immunology, Biological Sciences School, Universidad Autónoma de Zacatecas, Zacatecas, MEX; 2 Department of Internal Medicine, Hospital Regional Dr. Valentín Gómez Farías Instituto de Seguridad y Servicios Sociales de los Trabajadores del Estado (ISSSTE) Zapopan, Jalisco, MEX; 3 Department of Internal Medicine, Hospital Regional Elvia Carrillo Puerto Instituto de Seguridad y Servicios Sociales de los Trabajadores del Estado (ISSSTE) Merida, Yucatan, MEX; 4 Department of Dermatology, Hospital Civil de Culiacán Secretaria de Salud, Culiacan, MEX

**Keywords:** dermatillomania, excoriation disorder, neurotic excoriation, psychodermatosis, skin-picking disorder

## Abstract

Dermatillomania (DTM) is a psychodermatosis characterized by the presence of self-inflicted skin lesions due to excessive scratching in the absence of an underlying disease. We present the case of a 57-year-old female from a rural area of Zacatecas, Mexico, with an eight-year history of undiagnosed DTM, who exhibited extensive bilateral lesions on the arms, forearms, knees, and legs, which were successfully treated with sertraline alone. This article highlights the importance of DTM, its clinical presentation, and appropriate treatment to prevent complications.

## Introduction

Dermatillomania (DTM), also referred to as excoriation disorder or skin-picking disorder, is a mental health condition marked by the recurrent and uncontrollable urge to pick at one's own skin. This behavior can involve picking at healthy skin, blemishes, scabs, or any perceived skin imperfections. The repetitive nature of skin picking often results in noticeable tissue damage, including open sores, scarring, and infections, which can lead to significant physical discomfort and potential medical complications [1]. Beyond the physical consequences, DTM can cause substantial emotional and psychological distress. Individuals with this condition may experience feelings of shame, guilt, and embarrassment due to their skin-picking habits, leading to social isolation and reduced self-esteem. The disorder can also interfere with daily functioning, affecting work, school, and personal relationships [2]. Despite its significant impact on those affected, DTM remains a relatively misunderstood and under-researched condition within the broader field of mental health. Many individuals with DTM may not seek treatment due to a lack of awareness or the stigma associated with mental health disorders [3]. This document aims to provide a comprehensive overview of DTM, including its symptoms, causes, and treatment options. By shedding light on this disorder, we hope to increase awareness and understanding, ultimately fostering better support and care for those affected.

## Case presentation

A 57-year-old female from rural Zacatecas, Mexico, presented to our primary care clinic in April 2025 with an eight-year history of persistent dermatosis affecting her arms and legs. Her only significant medical history included well-managed type 2 diabetes, diagnosed 15 years prior, treated with metformin (850 mg three times daily) and NPH insulin (20 IU once daily in the morning). She received annual follow-up care through the endocrinology service.

The patient's chief complaint centered on chronic skin lesions accompanied by intense pruritus and an overwhelming urge to scratch. Despite previous interventions with systemic antibiotics and topical glucocorticoids by multiple physicians, her condition showed no improvement.

**Figure 1:**
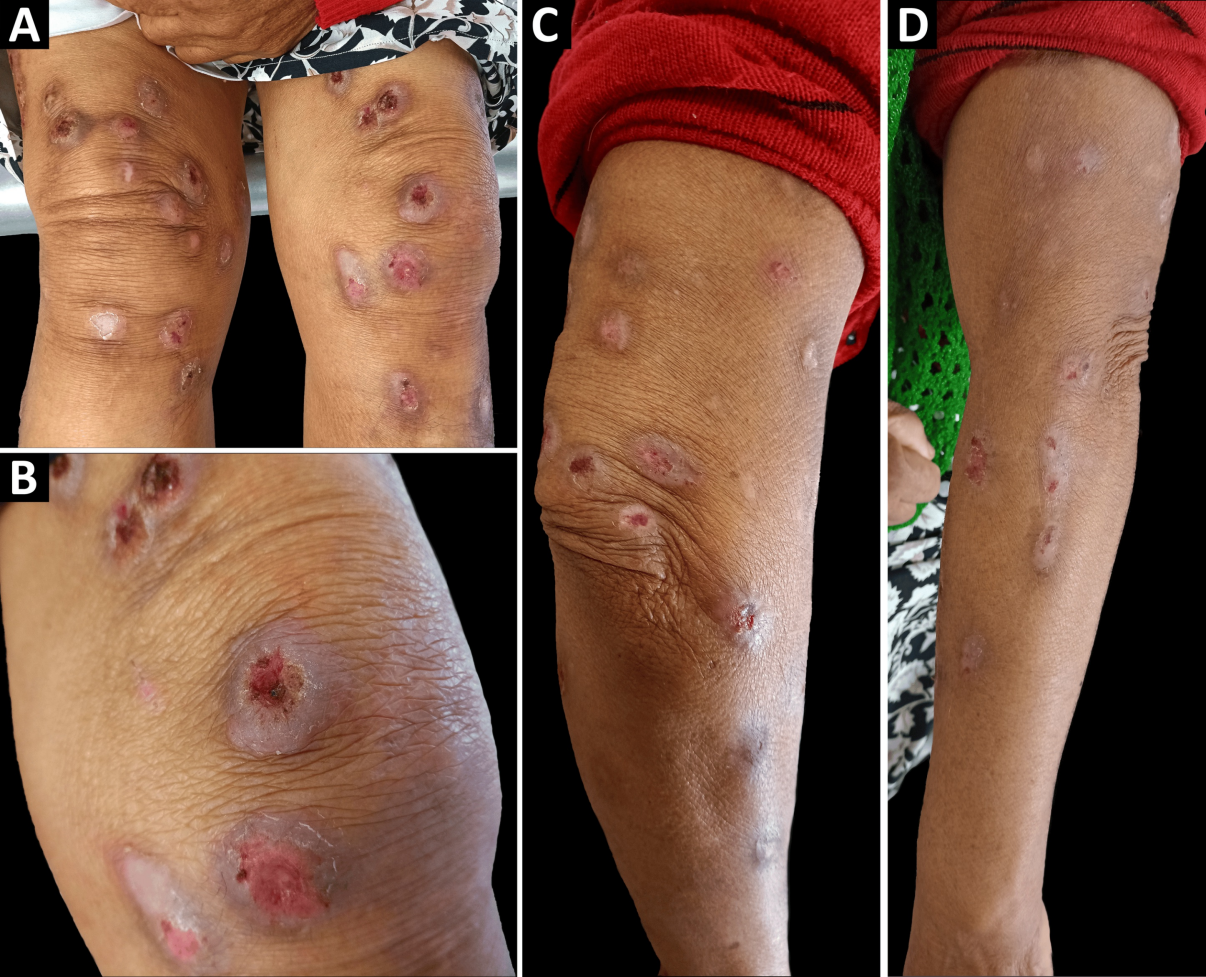
Psychogenic excoriation at initial evaluation The image shows multiple excoriations in different healing stages. (A) Front aspect of the knees. (B) A close-up picture of the left knee. (C) Posterior and lateral aspects of the right upper extremity. (D) Lateral aspect of the left upper extremity.

Physical examination revealed multiple bilateral oval excoriations, each approximately 1 cm in diameter. The lesions displayed characteristic features, including blood crusts surrounded by hypopigmented halos and scales. These excoriations were distributed symmetrically across the arms, forearms, knees, and legs (Figure 1). The lesions were painless but friable upon manipulation, with minimal bleeding. To ensure a comprehensive evaluation, we ordered a complete blood panel and obtained a skin biopsy for both culture and histopathological analysis, all of which returned normal results.

During the clinical interview, the patient provided crucial behavioral information, describing how she spent significant portions of her day engaged in scratching behaviors. She identified two primary triggers: physical pruritus and anxiety. The behavior was particularly prominent during sedentary activities, such as watching television, and before sleep. Once initiated, she reported considerable difficulty in stopping the scratching.

Based on the characteristic presentation, lesion distribution, patient history, and alignment with Diagnostic and Statistical Manual of Mental Disorders 5th edition (DSM-5) criteria, we established a diagnosis of DTM. Treatment was initiated with sertraline 50 mg daily, supplemented with urea cream for skin moisturization and hydroxyzine 25 mg three times daily for pruritus management. We employed the Skin Picking Scale - Revised (SPS-R) to establish a baseline severity score of 22 out of 32 [4].

**Figure 2:**
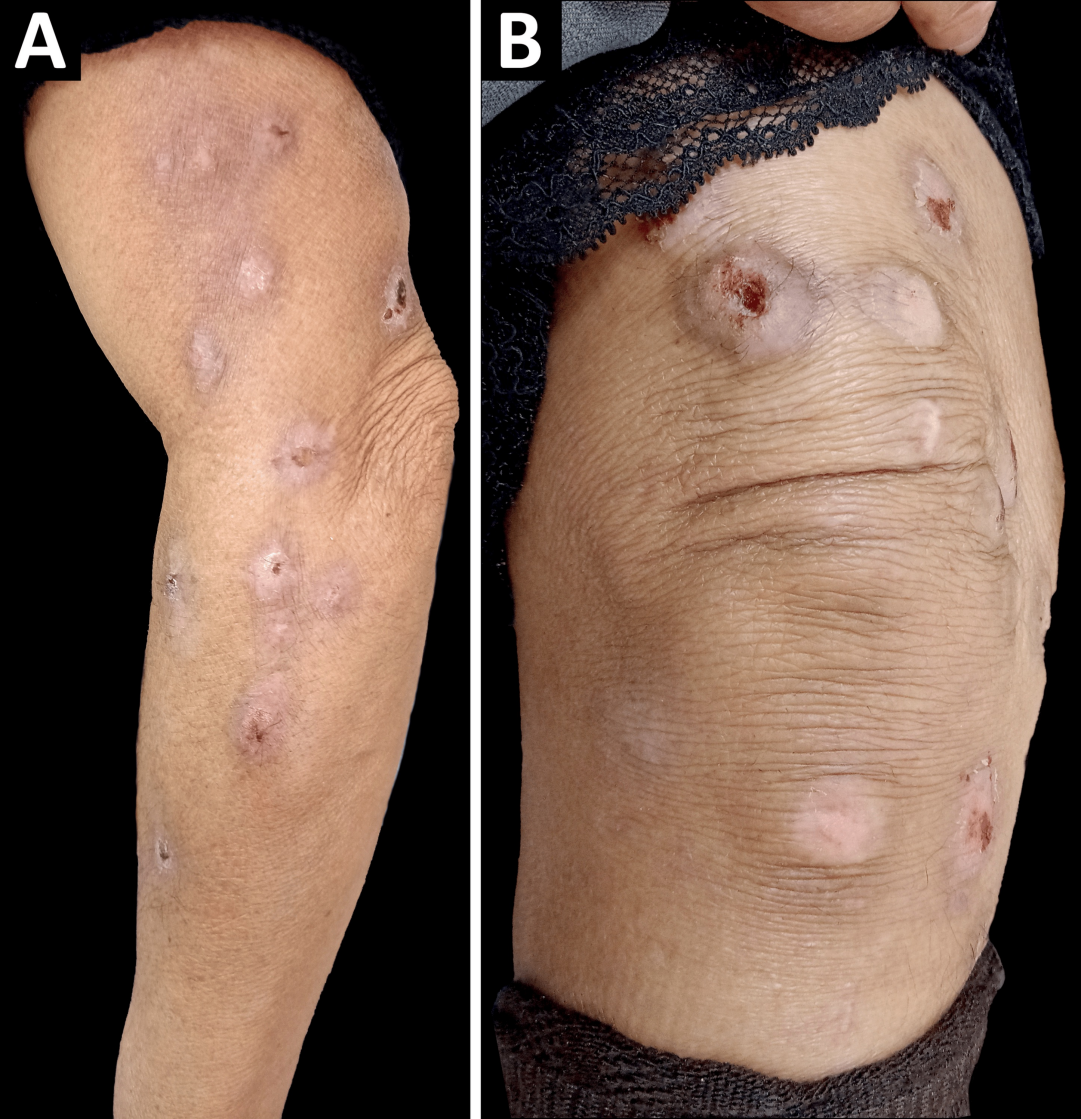
Psychogenic excoriation two months after treatment initiation The image shows a slight improvement in the lesions. (A) Lateral aspect of the upper extremity. (B) Front aspect of the right knee.

Due to geographical and financial constraints, the patient was unable to access specialized psychiatric care or cognitive-behavioral therapy (CBT) in the city. However, at the two-month follow-up, she demonstrated notable improvement, with visible reduction in lesion severity and scratching behavior and a decreased SPS-R score of 12 points (Figure 2). In view of the substantial clinical improvement in both symptomatology and cutaneous lesions, the therapeutic regimen was continued with sertraline as the primary pharmacological agent, supplemented by topical emollient cream for skin barrier maintenance, and first-generation antihistamines prescribed on an as-needed basis for episodes of severe pruritus. Bimonthly follow-up appointments were established to monitor lesion progression and serially assess disease severity using the SPS-R score. Throughout the treatment course, the patient has remained free of medication-related adverse effects and has demonstrated marked improvement in overall quality of life.

## Discussion

DTM belongs to the group of primary psychiatric skin disorders, which include delusional infestation, dermatitis artefacta, and trichotillomania, among others [1]. It is a psychodermatosis characterized by compulsive and repetitive self-scratching that causes skin excoriations [2]. It was first described by dermatologist Erasmus Wilson in 1875, who coined the term "neurotic excoriation" [5]. Since then, several names have been proposed to refer to this disease: excoriation disorder, skin picking disorder, psychogenic excoriation, and DTM [1,2]. In 2013, DTM was included in the obsessive-compulsive and related disorders section of the DSM-5 [2]. DTM affects an estimated 1-5% of the general population, though actual numbers may be higher due to underreporting [2]. A comprehensive systematic review by Farhat et al. (2023), analyzing 19 studies encompassing 38,038 patients, established a more precise prevalence of 3.5% (95% CI 2.55 to 4.65%) and demonstrated a gender disparity, with a female-to-male ratio of 1.5 [6]. The onset of DTM typically occurs during three distinct age periods: during adolescence, around age 21, and between ages 30 and 40 [1]. Without medical intervention, the condition follows a chronic course, characterized by periods of remission and exacerbation, with an average duration of 12 years [7]. The mean age of affected individuals is 33 years [6]. Several risk factors have been identified, including anxiety disorders, depression, obsessive-compulsive disorders (OCD), and low socioeconomic status [2].

Although the etiology of DTM is unknown, several studies have demonstrated its strong genetic association, with up to 29% of patients with DTM having first-degree relatives with some type of OCD, including DTM [8]. Our patient's case demonstrates both typical and atypical features of DTM. The presentation aligns with established literature regarding age of onset, female predominance, and chronic disease course, as evidenced by the eight-year duration. However, the case is notable for its departure from typical risk factor patterns, as our patient lacked the commonly associated psychiatric comorbidities, risk factors, or family history of OCD that are frequently reported in the literature.

Its clinical presentation is highly variable and is characterized by recurrent episodes of intense skin scratching, either with nails, fingers, teeth, or other objects (scissors, tweezers, among others). In order of frequency, lesions on multiple parts of the body are the most common; however, the face is the most affected area, followed by arms, forearms, chest, thighs, and feet. A characteristic feature of DTM is the so-called "butterfly sign," which refers to the upper and lateral area of the back being free of lesions due to its inaccessibility to the patient. Episodes are preceded by emotional stress, anxiety, boredom, and tension, among others, and scratching is triggered in response to a sensation of itching, burning, tingling, or dryness. Generally, episodes tend to be more frequent at bedtime, and patients typically experience a sense of gratification when scratching [1,2,9,10].

The severity of the lesions fluctuates over time due to periods of remission and exacerbation of symptoms over several years. Clinically, multiple skin lesions of approximately 1-3 cm in diameter can be observed in different stages of evolution: excoriations, bleeding, scars, crusts, ulcerations, and hypo- and hyperpigmented areas. Although it is not the rule, it is common to find other psychiatric disorders in these patients, especially anxiety, OCD, depression, and body dysmorphic disorder [2].

The diagnosis of DTM is established using DSM-5 diagnostic criteria, which encompasses several key elements. These include recurrent skin-picking behavior resulting in lesions, documented attempts by the patient to reduce or stop the behavior, significant impairment in social, occupational, or other important areas of functioning, and the absence of substance-induced effects or other mental disorders as the primary cause [3]. A crucial diagnostic feature that distinguishes DTM from other psychodermatoses is the patient's insight into the self-inflicted nature of their lesions and their expressed desire to cease the behavior [1]. This recognition of the behavioral component represents a critical differentiating factor in the diagnostic process.

Multiple scales have been proposed to determine the severity of the lesions and are mainly used to evaluate the effectiveness of treatment, with the SPS-R being one of the most used [4].

Our patient's presentation exemplifies classic DTM manifestations, characterized by typical lesions in readily accessible areas and notably demonstrating the characteristic "butterfly sign" sparing the upper back region. The pruritus-triggered behavior pattern and, most significantly, the patient's acknowledgment of self-induced lesions strongly supported the diagnosis. This insight particularly distinguishes DTM from other psychodermatoses, such as factitious dermatitis, where patients typically deny the self-inflicted nature of their lesions.

Treating DTM effectively often involves a team approach, including both a dermatologist and a psychiatrist [10]. Psychotherapy, particularly CBT, is the cornerstone of treatment, focusing on building awareness and managing habits [11]. However, when CBT isn't enough, medications can be added to the treatment plan. Selective serotonin reuptake inhibitors (SSRIs) like sertraline and fluoxetine are commonly prescribed to help reduce compulsive and anxious behaviors by increasing serotonin levels [12]. Naltrexone, an opioid antagonist, can also be used to decrease the satisfaction associated with scratching by blocking opioid receptors [12,13]. Additionally, N-acetylcysteine, a precursor of glutathione and modulator of the neurotransmitter glutamate, widely known for its effects as a mucolytic and antidote for acetaminophen poisoning, has shown effectiveness in reducing compulsive behavior in patients with DTM by attenuating glutamatergic hyperactivity, releasing glutamate (the main excitatory neurotransmitter) to the central nervous system [14]. Other medications, such as tricyclic antidepressants, antipsychotics, antiepileptics, and lithium, can be considered as second-line options. Some patients also find relief through alternative therapies like meditation, acupuncture, yoga, and hypnosis [2,15]. In the presented case, the patient's limited financial resources led to a treatment approach using only an SSRI along with an antihistamine to manage itching, which proved effective in lowering the severity of the condition.

## Conclusions

DTM is a significant psychodermatological condition characterized by compulsive skin picking, leading to various physical and psychological challenges. The case presented highlights the complexities of diagnosing and managing DTM, especially in patients with limited access to specialized psychiatric care. The successful treatment of a 57-year-old female with sertraline underscores the potential of SSRIs in managing DTM symptoms, even in the absence of comprehensive psychotherapy. The patient's improvement, as indicated by the reduction in the SPS-R score, demonstrates the effectiveness of targeted pharmacological intervention in alleviating compulsive behaviors and reducing the severity of skin lesions. This paper emphasizes the importance of recognizing DTM as a distinct clinical entity and implementing appropriate treatment strategies to mitigate its impact on affected individuals. Early diagnosis and intervention can prevent the progression of the disorder, reduce the risk of complications, and improve the overall quality of life for patients with DTM. Further research is needed to explore the underlying mechanisms of DTM and develop more effective and accessible treatment options for diverse patient populations. A multidisciplinary approach involving dermatologists, psychiatrists, and other healthcare professionals is essential to provide comprehensive care and support for individuals struggling with DTM.
